# Prevalence of non-syndromic orofacial clefts: based on 15,094,978 Chinese perinatal infants

**DOI:** 10.18632/oncotarget.24238

**Published:** 2018-01-13

**Authors:** Dazhi Fan, Shuzhen Wu, Li Liu, Qing Xia, Guo Tian, Wen Wang, Shaoxin Ye, Lijuan Wang, Jiaming Rao, Xiao Yang, Zhen Yu, Lihong Xin, Song Li, Zhenghua Duan, Tianchen Zhang, Song Wu, Xiaoling Guo, Zhengping Liu

**Affiliations:** ^1^ Foshan Institute of Fetal Medicine, Southern Medical University Affiliated Maternal and Child Health Hospital of Foshan, Foshan, Guangdong, 528000, China; ^2^ Department of Obstetrics, Southern Medical University Affiliated Maternal and Child Health Hospital of Foshan, Foshan, Guangdong, 528000, China; ^3^ Department of Epidemiology and Biostatistics, School of Public Health, Anhui Medical University, Hefei, Anhui, 230032, China; ^4^ School of Integrated Traditional and Western Medicine, Anhui University of Chinese Medicine, Hefei, Anhui, 230038, China; ^5^ Department of Library, The First Affiliated Hospital, College of Medicine, Zhejiang University, Hangzhou, Zhejiang, 310003, China; ^6^ Menzies Institute for Medical Research, University of Tasmania, Private Bag 23, Hobart, Tasmania 7000, Australia; ^7^ Department of Epidemiology, Medical College of Jinan University, Guangzhou, Guangdong, 510632, China; ^8^ Changzhou Center for Disease Control and Prevention, Changzhou, Jiangsu, 213003, China; ^9^ Department of Maternal, Child and Adolescent Health, School of Public Health, Anhui Medical University, Hefei, Anhui, 230032, China; ^10^ Department of Neurology, The Second Affiliated Hospital of Soochow University, Suzhou, Jiangsu 215004, China; ^11^ Chaohu Hospital Affiliated Anhui Medical University, Chaohu, Anhui, 238000, China; ^12^ Chengdu Center for Disease Control and Prevention, Chengdu, Sichuan, 610041, China; ^13^ Department of Communicable Diseases Control, Jiangxi Provincial Center for Disease Control and Prevention, Nanchang 330000, Jiangxi, China

**Keywords:** non-syndromic orofacial clefts, perinatal infants, prevalence, meta-analysis, China

## Abstract

Non-syndromic orofacial clefts (NSOFC), which include cleft lip and palate (CLP), cleft lip only (CLO), and cleft palate only (CPO), contains a range of disorders affecting the lips and oral cavity. No systematic review and meta-analysis has been carried out to synthesize the prevalence of NSOFC in Chinese perinatal infants. We aimed to quantify and understand the variation of prevalence national and regional levels. Four English databases and four Chinese databases were searched using a comprehensive search strategy from inception to April 2017. The random effect model was used for this meta-analysis. To determine the sources of heterogeneity, subgroup analyses and meta-regression were conducted based on different categories. The protocol has been pre-registered in the PROSPERO, number CRD42017062293. 110 studies, including 15,094,978 Chinese perinatal infants, were eligible for inclusion. The pooled prevalence rate for NSOFC was 1.67‰ (95% CI 1.53–1.82), varying with provinces. The pooled prevalence estimate was 0.56‰ (0.50–0.63) for CLO, 0.82‰ (0.73–0.90) for CLP, and 0.27‰ (0.24–0.30) for CPO. Significant associations were found between overall prevalence estimates and survey year and study region. The prevalence of NSOFC was severe in Chinese perinatal infants, varying with provinces. The results will serve as a baseline for future assessment of the overall effectiveness of NSOFC control, and will also support and inform health policy for planning and helping health debates.

## INTRODUCTION

Non-syndromic orofacial clefts (NSOFC), which include cleft lip and palate (CLP), cleft lip only (CLO), and cleft palate only (CPO), contains a range of disorders affecting the lips and oral cavity [1, 2]. The etiology and pathogenesis of NSOFC remain largely unknown and involve both genetic and environmental factors contributing to the phenotype [3–5]. It has been shown that these congenital disorders have a significant negative impact on audition, speech, appearance and psychology, which could affect the individuals and their families [6]. Theses impacts can lead to considerable adverse health outcomes and enormous socioeconomic burden [7]. The global average prevalence of NSOFC was approximately 1.7‰ in live birth babies [1]. However, the prevalence of NSOFC varies broadly with the difference of ethnicities and geographical positions, and populations of African ancestry having the lowest rates while groups of Amerindian and Asian ancestry possessing the largest [5]. One previous systematic review and meta-analysis emerged in the scientific literature found that the birth prevalence of orofacial clefts was 1.38 per 1000 birth in low- and middle-income countries [8].

China is the biggest developing country and has the largest population in the world. Since mid-1980s, congenital anomalies surveillance systems have been progressively established in hospitals in mainland China [9]. After 30-year effort, all the infants (including live births, stillbirths, abortion or dead fetus) during perinatal period (between 28 gestation weeks and 7 days after birth) will be registered in hospitals. Individual studies have demonstrated that NSOFC (including CLO, CLP, and CPO) is the most common congenital disorders in Chinese perinatal infants [10–12]. Nevertheless, estimates of the prevalence of NSOFC among perinatal infants vary across studies from 0.32‰ to 4.70‰ [13, 14].

Reliable estimate of prevalence is important for informing efforts to prevent, treat, and identify causes of this disorder among perinatal infants. Hence, the relevant studies are warranted. Meanwhile, many factors, such as survey year, hospital level, sample size, and geographical locations could easily influence the results. We, therefore, aimed to address this research gap by means of a systematic review and meta-analysis of published studies. To the best of our knowledge, this is the first study to determine the prevalence and the characteristics of NSOFC (including CLO, CLP, and CPO) in Chinese perinatal infants. The results will be essential for policymakers and health professionals aware of this influential congenital disorder in Chinese perinatal infants and will be useful for the planning of health services.

## RESULTS

### Characteristics of study

**Figure 1:**
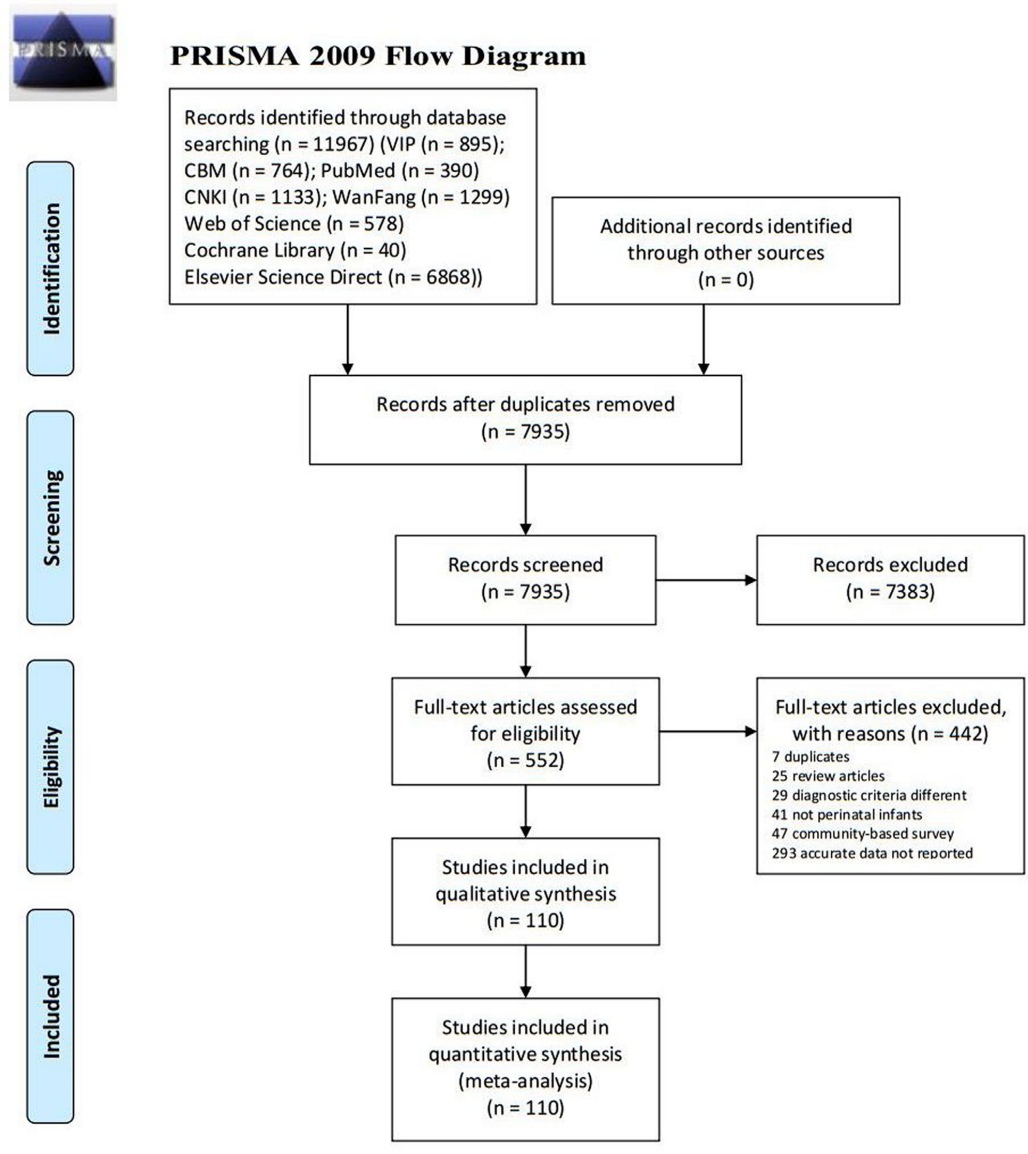
Flowchart of study selection

In total, 11967 studies were identified using the initial literature search. The search results were as follows: PubMed (*n* = 390), Elsevier Science Direct (*n* = 6868), Web of Science (*n* = 578), Cochrane Library (*n* = 40), CBM (*n* = 764), CNKI (*n* = 1133), Chongqing VIP (*n* = 895), and WanFang (*n* = 1299). After removing 4032 duplicates and 7383 apparently irrelevant citations by title and abstract review, 552 potentially eligible articles and were reviewed in full-text level. After carefully reading, 442 of them were excluded for various reasons (including 7 duplicates, 25 review articles, 29 diagnostic criteria different, 41 not perinatal infants, 47 community-based survey and 293 accurate data not reported). Finally, a total of 110 articles, including 138 survey researches, met the inclusion criteria in the final meta-analysis (Figure 1).

Detailed characteristics of each included study were shown in Supplementary Table 1. Twenty-nine studies with regarding to the prevalence of NSOFC in Chinese perinatal infants were implemented in East China (4 in Anhui, 2 in Fujian, 5 in Jiangsu, 3 in Jiangxi, 6 in Shandong, 3 in Shanghai, 6 in Zhejiang), 12 in North China (2 in Beijing, 2 in Hebei, 2 in Inner Mongolia, 3 in Shanxi, 3 in Tianjin), 16 in Northwest China (2 in Gansu, 6 in Ningxia, 2 in Qinghai, 3 in Shaanxi, 3 in Xinjiang), 16 in South China (11 in Guangdong, 4 in Guangxi, 1 in Hainan), 13 in Central China (3 in Henan, 3 in Hubei, 7 in Hunan), 10 in Northeast China (2 in Heilongjiang, 4 in Jilin, 4 in Liaoning), and 10 in Southwest China (1 in Chongqing, 2 in Guizhou, 2 in Sichuan, 1 in Tibet, 4 in Yunnan).

A total of 110 articles including 15,094,978 Chinese perinatal infants with a mean number of 137,227 were included. The sample sizes of the included articles ranged from 4192 to 1395155. The articles were published from 1989 to 2017. Of the 110 eligible articles, the mean NOS score was 7.83, with 24 studies scored 9, 45 studies scored 8, 39 studies scored 7, and 2 studies scored 6.

### Meta-analysis

Seventy-eight articles, which were constituted of 106 surveys, have reported the prevalence of NSOFC based on the data of 9476601 Chinese perinatal infants. The prevalence of NSOFC varied from 0.32‰ (95% confidence interval [CI], 0.28–0.35) [13] to 4.70‰ (95% CI, 3.45–5.95) [14]. The meta-analysis revealed significantly high heterogeneity across studies (*I*^2^ = 97.3%, *p* < 0.001). Using a random effects model, the pooled point prevalence of NSOFC was 1.67‰ (95% CI, 1.53–1.82; *I*^2^ = 97.3%). Sensitivity analysis showed that the pooled prevalence was between 1.66‰ (95% CI, 1.51–1.80) (after excluding Zhang JX et al. [14]) and 1.69‰ (95% CI, 1.54 – 1.83) (after excluding Yi YT et al. [15]). These similar results indicated that no individual study affected the overall pooled estimate of the meta-analysis. As shown in Supplemenatry Figure 1, although the funnel plot was slightly asymmetrical, no publication bias was found according to the results of both Egger’s test (t = −0.21, *p* = 0.834) and Begg’s test (z = 1.94, *p* = 0.053).

### Subgroup analyses

We further examined pooled prevalence according to the subtypes of CLO, CLP, and CPO. For the 92 studies that included 7511 perinatal infants with CLO, the pooled prevalence was 0.56‰ (95% CI, 0.50 – 0.63). Another 84 studies that included 8463 perinatal infants with CLP gave a pooled prevalence of 0.82‰ (95% CI, 0.73 – 0.90), and a pooled prevalence of 0.27‰ (95% CI, 0.24 – 0.30) were calculated from 42 studies, including 1959 perinatal infants with CPO.

**Figure 2:**
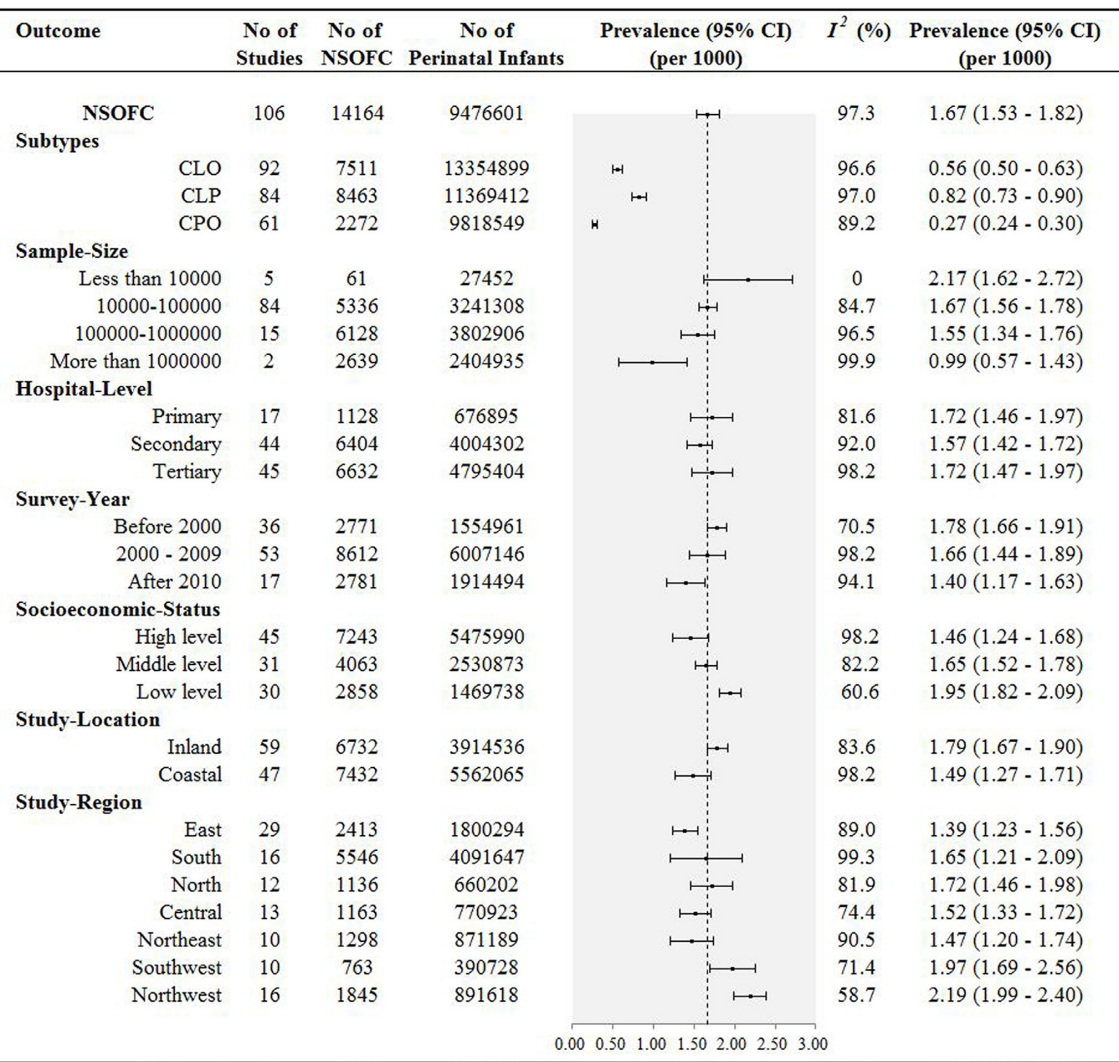
Results of subgroup analysis for the pooled prevalence of NSOFC in Chinese perinatal infants

To further characterize the potential impact to the pooled prevalence from the different sample sizes of the included studies, meta-analysis was stratified by sample size. Pooled prevalence estimates were: 0.99‰ (95% CI, 0.57–1.43) for sample sizes more than 1000000 individuals; 1.55‰ (95% CI, 1.34–1.76) for sample sizes between 100000 and 1000000; 1.67‰ (95% CI, 1.56–1.78) for sample sizes between 10000 and 100000; and 2.17‰ (95% CI, 1.62–2.72) for sample sizes less than 10000 (Figure 2).

**Figure 3:**
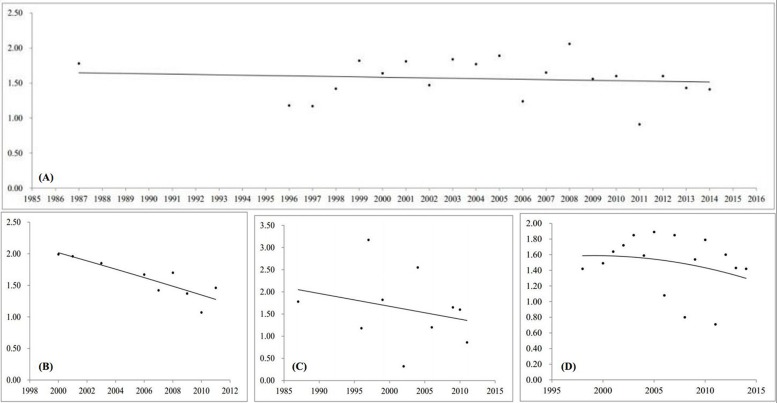
Trend lines of the prevalence of NSOFC over the year (x axes is the survey year, y axes is prevalence (per 1000)) (**A**) all of Chinese perinatal infants, (**B**) by primary hospital, (**C**) by secondary hospital, (**D**) by tertiary hospital.

According to hospital level, the results were classified as follows: primary hospital (1.72‰, 95% CI, 1.46–1.97), secondary hospital (1.57‰, 95% CI, 1.42–1.72), and tertiary hospital (1.72‰, 95% CI, 1.47–1.97). Prevalence of NSOFC by year groups of before 2000, 2000–2009, and 2010 to date were 1.78‰ (95% CI, 1.66 –1.91), 1.66‰ (95% CI, 1.44–1.89), and 1.40‰ (95% CI, 1.17–1.63), respectively (Figure 2). Although there were no significant time trends, the trend line showed that there was a progressively decreasing of prevalence over the year. The decreasing was also reflected in each hospital level, including primary-, secondary- and tertiary-hospital (Figure 3). In subgroup analysis based on socioeconomic status, the prevalence was highest in low level socioeconomic condition (1.95‰, 95% CI, 1.82–2.09), followed by middle level (1.65‰, 95% CI, 1.52–1.78), and high level (1.46‰, 95% CI, 1.24–1.68).

**Figure 4:**
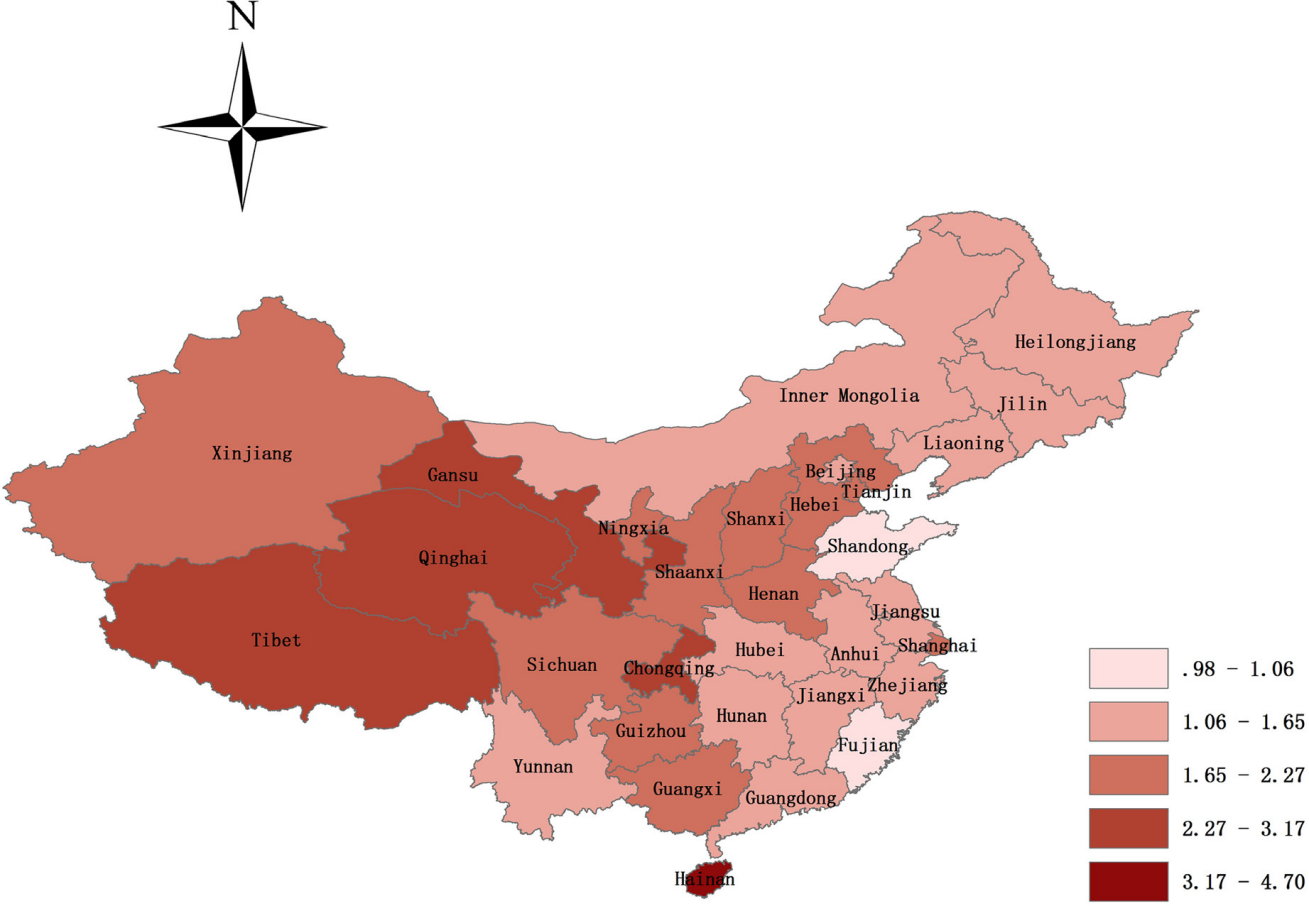
Map of the prevalence of NSOFC in Mainland Chinese perinatal infants (not including Hong Kong, Macao, and Taiwan) The pooled prevalence of NSOFC among perinatal infants was 4.70‰ in Hainan, followed by 3.17‰ in Chongqing, 3.00‰ in Qinghai, 2.84‰ in Tibet, 2.46‰ in Gansu, 2.27‰ in Guizhou, 2.18‰ in Ningxia, 2.11‰ in Shaanxi, 1.97‰ in Shanxi and Sichuan, 1.94‰ in Henan, 1.80‰ in Xinjiang, 1.78‰ in Hebei, 1.77‰ in Shanghai, 1.74‰ in Tianjin, 1.72‰ in Guangxi, 1.65‰ in Anhui, 1.62‰ in Jilin, 1.57‰ in Zhejiang, 1.55‰ in Jiangxi, 1.54‰ in Inner Mongolia, 1.53‰ in Yunnan, 1.51‰ in Heilongjiang, 1.45‰ in Hunan, 1.44‰ in Guangdong, 1.44‰ in Hubei, 1.37‰ in Jiangsu, 1.35‰ in Beijing, 1.29‰ in Liaoning, 1.06‰ in Fujian, and the lowest is 0.98‰ in Shandong.

Regarding different study location, fifty-nine and forty-seven studies offered the prevalence of NSOFC in inland and coastal area, respectively. The prevalence was 1.79‰ (95% CI, 1.67–1.90) in inland area perinatal infants, and coastal area perinatal infants seemed to have a lower prevalence with an estimation of 1.49‰ (95% CI, 1.27–1.71). Among study regions, the highest prevalence was 2.19‰ (95% CI, 1.99–2.40) in northwest China, followed by southwest China (1.97‰ (95% CI, 1.69–2.56)), then north China (1.72‰ (95% CI, 1.46–1.98)), south China (1.65‰ (95% CI, 1.21–2.09)), central China (1.52‰ (95% CI, 1.33–1.72)), and northeast China (1.47‰ (95% CI, 1.20–1.74)), and east China had the lowest prevalence (1.39‰ (95% CI, 1.23–1.56)). Further subgroup analysis found that the highest pooled prevalence of NSOFC among perinatal infants was 4.70‰ in Hainan, and the lowest was 0.98‰ in Shandong. There were significant varied with the provinces (Figure 4).

Sample size, survey year, hospital level, and study region were entered into the meta-regression, which yielded a significant model (F = 4.11, *p* = 0.0040) that explained 13.59% of the variance. Meanwhile, we found that survey year and study region were significantly associated with heterogeneity in this meta-analysis. The t-values were 2.27 and -2.58; and the *p*-values were 0.025 and 0.011 for the two variables, respectively.

## DISCUSSION

To the best of our knowledge, this is the first systematic review and meta-analysis based on Chinese hospital-based congenital anomalies surveillance systems to determine the national prevalence and its characteristics of non-syndromic orofacial clefts (NSOFC). The present systematic review included 110 articles, involving 15,094,978 Chinese perinatal infants in all 31 provinces, to demonstrate the prevalence of NSOFC and its subgroups, cleft lip only (CLO), cleft lip and palate (CLP), and cleft palate only (CPO). The prevalence of NSOFC was 1.67‰ (95% CI, 1.53–1.82). Further results showed that prevalence estimates for CLP was highest (0.82‰), followed by CLO (0.56‰) and CPO (0.27‰). Given the approximate 2016 population estimates of 17 million perinatal infants [16], this rate amounts to 28390 suffers of NSOFC annually in China.

NSOFC is one of the most common congenital birth defects in perinatal infants and imposes a substantial physical and financial burden on affected individuals and families [3, 6]. Fetuses with NSOFC usually could be identified by ultrasound. Prenatal transabdominal sonographic could detect the defects during the second trimester (14–28 weeks) of pregnancy [17]. The detection rate can reach 100% [18], however, the accuracy is highly variable and dependent on the experience of the sonographer, gestational age, the types and fetal position [19]. The sex ratio (male : female) was reported about 2:1 in white population [1] and about 1.25:1 in Chinese population [20]. About seven in ten (68%) NSOFC infants were suffered from malnutrition, which is also a major cause of the high mortality of infants [21].

NSOFC is generally classified as CLP, CLO, or CPO. Because CPO is less noticeable externally, the prevalence of CPO might underestimate in perinatal infants in initial survey studies. Findings from this study showed that the prevalence of CPO accounted for only a half of CLO and one third of CLP in Chinese perinatal infants. Meanwhile, previous studies [6, 8] were also found CLP high compared to CLO, which were similar to our result. A possible explanation for this difference is that cleft hard palate is often accompanied by a cleft lip [1]. Furthermore, difficulties in identifying perinatal infants with not obvious cleft lip are likely to lead to underascertainment at the beginning of the studies [22].

We found that the prevalence of NSOFC reduced gradually from 2.17 to 0.99 per 1000 perinatal infants with the increasing of sample size, which plays an important role in epidemiology surveys. Small sample sizes are more likely to lead to instable results, especially in the calculation of prevalence in rare diseases [23]. Systematic review and meta-analysis provides a scientific and logical method to synthesize epidemiological data, and it could enhance the statistical power as well as draw a more reliable conclusion when compared with single studies [24].

A recent systematic review showed that the overall prevalence of orofacial clefts was 1.40‰ in Chinese live births [25]. Because few included studies reported relevant data, the study was not estimated the prevalence of cleft types (CLO, CLP, and CPO). And more importantly, Mossey P has reported that stillbirths have higher prevalence of orofacial clefts than live births [26]. Thus, the prevalence rate of orofacial clefts in live births is generally lower than in perinatal infants. Our results further confirmed Mossey P’s findings. Because of the difference in participants, the results, including region, socioeconomic status, sample size, will be different between the two articles.

Genetic and epidemiological studies have clarified that the etiology of NSOFC is involving genetic and environmental risk factors [27–30]. Findings of genome-wide and genome-wide meta-analyses have suggested various loci could have a causal role in NSOFC, including 1p22.1: rs560426, 1q32.2: rs861020, 8q24: rs987525, 10q25: rs7078160, and 13q31.1: rs8001641 [28, 29]. About 30% NSOFC cases can be contributed to these various locus [1, 28, 29]. In addition, epidemiological and experimental data suggested that poor nutrition, maternal tobacco smoking, maternal alcohol use, viral infection, and glucocorticoid exposure were risk factors for NSOFC [31–33]. Maternal smoking during pregnancy has been associated with increased risk of NSOFC, with a population-attributable risk as high as 20% [34, 35].

Stratified by time spans, the highest prevalence of NSOFC was observed before 2000, and the lowest in 2010 to date. Although the results were not significantly different, it seems that there is a decreasing trend of prevalence with time. After further study, the trend line also showed there was a progressively decreasing of prevalence over the year. On one hand, the high prevalence appeared before 2009 might be partly explained by complete ascertainment through the registry and improved prenatal detection [36]. On the other hand, the decreasing trend after 2010 might be attributed that the government refocused the importance of the primary prevention for women at childbearing age [37]. Most cities [38–40] have switched from prenatal care to preconceptional or periconceptional care through the Local Family Planning System and Maternal and Child Health Care System in recent years. In addition, prenatal diagnostic techniques, including fetal ultrasound, fetal echocardiography, and karyotyping following amniocentesis and chorionic villus sampling have been available to diagnose severe structural defects before early second trimester (usually before 22 gestational weeks) [41], and virtually all mothers who carry a fetus affected by a severe malformation would choose elective termination [42].

China is a vast, multiracial country, and in this study we found significant differences in the prevalence of NSOFC in perinatal infants by territories (inland vs coastal, and between regions and provinces). Inland perinatal infants have a higher prevalence compared with that of coastal area, and prevalence varied substantially among the 7 regions and 31 provinces. The prevalence of provinces spans from 0.98 to 4.70 per 1000 perinatal infants. The highest reported rates were in Hainan, Chongqing, and Qinghai, with prevalence rates of 4.70, 3.17, and 3.00 per 1000 perinatal infants, respectively. The lowest reported rates were in Shandong, Fujian, and Liaoning, with prevalence of 0.98, 1.06, and 1.29 per 1000 perinatal infants, respectively. It is likely that many factors could contribute to this geographic difference, such as environmental pollution, economic status, health service status, and diagnostic level [11].

Most of the minorities are concentrated in northwest and southwest developing provinces in China [43]. Compared to east developed provinces, there are some differences in their genetics and lifestyle [44, 45]. Meanwhile, prenatal diagnostic techniques vary greatly among provinces and hospitals in China. The developed provinces have made the greatest efforts in prenatal screening and diagnosis and mothers would choose elective termination before 22 gestational weeks when they found a fetus affected by a severe malformation [42]. Meanwhile, most coastal cities attached importance to the primary prevention in childbearing age women, which could reduce the occurrence of birth defects [39]. The above reasons can be partially explained why there is the different prevalence based on different regions. To reduce the incidence of NSOFC, preconception care and antenatal screening should be promoted in every women of reproductive age.

There are many strengths in our systematic review and meta-analysis. No systematic review and meta-analysis has been performed to synthesize the prevalence of NSOFC in Chinese perinatal infants. Our study is the first to adhere to PRISMA guidelines to quantify prevalence estimates derived from a comprehensive search strategy. It has a relatively large number of perinatal infants which including 15,094,978 across all 31 provinces in mainland China, and this allows us to get a stable result. Meanwhile, overall quality of the included studies was acceptable, publication bias tests did not show potential risk, and sensitivity analysis was not substantially altered.

Despite the strengths of this study, several limitations need to be considered in interpreting with future research. On one hand, like other similar prevalence meta-analyses [46–49], heterogeneity usually existed, and was not fully resolved by subgroup and regression model, although survey year and study region could explain a part of its heterogeneity. On the other hand, it should be noted that due to mild disorders, especially in CPO, undiagnosed in utero or lack of external visibility at birth may be underestimate the true prevalence. Meanwhile, the sources of data were all captured from hospital-based surveillance systems, which could also underestimate the prevalence compared with population-based survey. In addition, because of the limited information in included studies, this prevalence meta-analysis could not provide more detailed results, such as age of infants when disease was diagnosed, sex ratio of infants diagnosed with NSOFC, and trend lines of prevalence in each province.

In this systematic review, the pooled estimate of the prevalence of NSOFC was 1.67 per 1000 in Chinese perinatal infants, varying with provinces. The result will serve as a baseline for future assessment of the overall effectiveness of the NSOFC control in China, and will also could support and inform health policy for planning and helping health debates.

## MATERIALS AND METHODS

### Protocol and registration

Similar as our previous studies [50–52], this meta-analysis was also strictly performed according to the guidelines in the Preferred Reporting Items for Systematic Reviews and Meta-Analyses (PRISMA) statement (Supplementary Table 2) [53]. The protocol for this systematic review was pre-registered in the PROSPERO International Prospective Register of systematic reviews (http://www.crd.york.ac.uk/PROSPERO/), and the registration number is CRD42017062293.

### Search strategy

Data for this meta-analysis was comprehensively identified by searching four English databases (PubMed, Elsevier Science Direct, Web of Science, Cochrane Library) and four Chinese databases (Chinese Biological Medical Literature database (CBM), Chinese National Knowledge Infrastructure (CNKI), Chongqing VIP (VIP), WanFang) from inception to April 2017. Search strategy was listed as follows: (“birth defect” OR “orofacial clefts” OR “cleft lip” OR “cleft lip and palate” OR “cleft palate”) AND (“prevalence” OR “incidence” OR “epidemiology” OR “Survey”) AND (“China” OR “Chinese”). In addition, snowball searching of reference lists was also conducted to find further relevant articles.

Language was restricted to either Chinese or English. Two researchers (L Liu and G Tian) independently reviewed all titles and abstracts. The complete relevant articles were downloaded for further screening. If the same data was reported in more than one publication, only the paper with a better quality was included. Any uncertainty was settled by discussion with the third participant (D Fan). In addition, we performed a further search by perusing the references of review articles and e-mailing the authors for full-text articles to include all available data in this meta-analysis.

### Inclusion and exclusion criteria

Articles fulfilling the following criteria were considered eligible for inclusion: 1) cross-sectional studies examining prevalence of NSOFC (including CLO, CLP, and/or CPO) in perinatal infants (between 28 weeks of gestation and 7 days after birth) in mainland China (not including Hong Kong, Taiwan, and Macao); 2) the diagnosis was based on the Chinese national criteria of birth defects [9]; 3) sample sizes from hospital-based survey; 4) publications in full text either written in English or in Chinese. Studies conducted in specific populations (e.g., children, adolescents, and the elderly) or special settings (e.g. community-based, population-based) and studies used census sampling were excluded.

### Data extraction

The available data extraction was independently performed by two researchers using a standardized form, and the disagreement was resolved through discussion. The key data extraction sheet included: first author, year of publication, year of the study conducted, province where the survey was conducted, number of perinatal infants, number of NSOFC (CLO, CLP, and/or CPO).

### Quality assessment

In line with other and our previously studies [54, 55], two researchers (SZ Wu and W Wang) independently assessed the risk of bias and methodological quality of each study using the Newcastle-Ottawa Score (NOS) for evaluating the quality of observational epidemiological studies [56].

### Statistical analysis

In this meta-analysis, all statistical analyses were undertaken with STATA software, Version 12.0 (Stata Corporation, College Station, Texas, USA). Cochran’s Q statistic and *I*^2^ index were calculated to assess the heterogeneity of the prevalence across studies. A p < 0.05 or the *I*^2^ statistic > 50% indicated heterogeneity in the effect size [57]. Due to heterogeneity across studies, a random effect model was used for this meta-analysis [58]. To determine the sources of heterogeneity, subgroup analyses and meta-regression were conducted based on different categories: sample size (< 10000, 10000 - 100000, 100000 – 1000000, and > 1000000), year of data collection (before 2000, 2000–2009, and after 2010), hospital level (primary, secondary, and tertiary), socioeconomic status [59], study location (inland and coastal), and study region (east, north, northwest, northeast, southwest, central, and south). Moreover, in order to more clearly display area distribution, the prevalence of each province was also calculated. Maps were drawn using ArcMap version 10.2 (Environmental Systems Research Institute, Redlands, CA). To test the robustness of the result on the overall prevalence estimates, sensitivity analysis was performed by serially excluding each included studies. Publication bias was estimated by testing for funnel plot asymmetry and using Egger’s and Begg’s test. All *p*-values were two-sided, and *p* < 0.05 was regarded statistically significant.

## SUPPLEMENTARY MATERIALS FIGURE AND TABLES






