# Supplementary material for: Prevalence of non-syndromic orofacial clefts: based on 15,094,978 Chinese perinatal infants
Source: Oncotarget. 2018 Jan 13;9(17):13981–90. doi: 10.18632/oncotarget.24238 (PMC5862631; doi:10.18632/oncotarget.24238)
Supplement: Supplementary file 1 [file oncotarget-09-13981-s001.pdf]

Prevalence of non-syndromic orofacial clefts: based on 15,094,978 Chinese perinatal infants

SUPPLEMENTARY MATERIALS

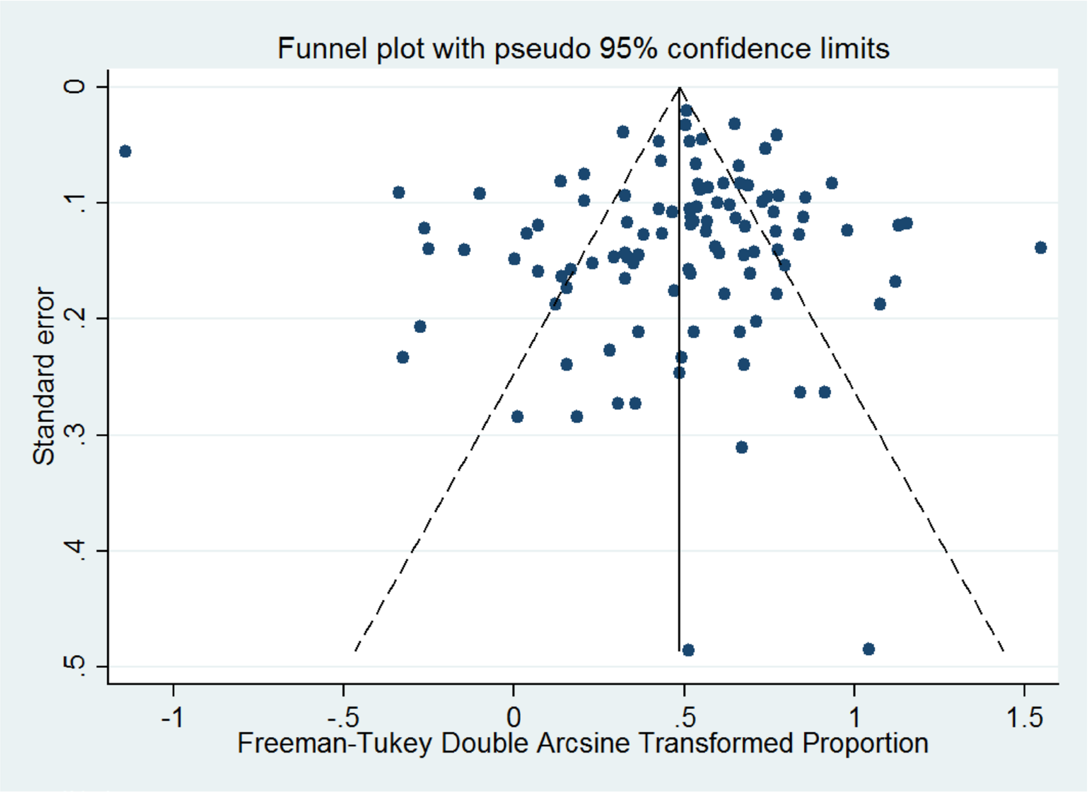

Supplementary Figure 1: Funnel plot of the studies included in the NSOFC meta-analysis.

Supplementary Table 1: Detailed characteristics of the included studies. See_Supplemenatry_Table 1.

Supplementary Table 2: PRISMA 2009 Checklist. See_Supplemenatry_Table 2.
